# Ultrasound virtual skills based workshop: An African experience in the COVID era

**DOI:** 10.1016/j.afjem.2021.10.004

**Published:** 2021-12-28

**Authors:** R. Wahome, D. Mirsch, L. Kariuki, B. Monaco, K. Bagonza, D. Harborne

**Affiliations:** a Mbarara University of Science and Technology, Uganda; b University at Buffalo New York, United States of America

**Keywords:** Telemedicine, Ultrasound, Mentorship, Skills, Low-resource

## Abstract

Telemedicine has emerged as a valuable tool for medical training, now more than ever. It involves exchanging healthcare or healthcare information digitally across large distances. This form of teaching has become more common due to significant advances in communication technology and increased access to the internet at more affordable costs. Isolated and poorly staffed areas are now able to access specialist review, mentorship, educational materials, and general support more efficiently than before. Typically, telemedicine is used to deliver didactic sessions and lectures and not skill sharing or training exercises. While ultrasonography is a skill typically taught at the bedside, we face a global pandemic where patient safety and standard operating procedures are prohibitive of this teaching model. Our team sought to have a practical session to determine whether practical skills can be taught through virtual training workshops as a way to mitigate these constraints. Practical stations were set up, with each station hosting an independent skill. The aim of the session was to introduce the topics to learners, to have learners visualize how the scans can be done with local setup and lastly perform these scans on volunteers to the satisfaction of the supervisors. Skills such as performing ocular ultrasound, gallbladder evaluation, and aortic aneurysm assessment were carried out on volunteers under a virtual supervisor's direction at all stations. The topics were chosen based on a previous needs assessment, and participants reported great satisfaction from the session. Ultrasound provides an excellent opportunity for virtual skill-based training, mentorship and trainee support. This commentary is directed at mostly low resource African countries with nascent Emergency Medicine programs. It also applies to organizations that support remote ultrasound skills training for emergency care providers and those that run emergency care outreach programs. These principles may also apply for other lower resource settings outside of Africa.

## African relevance

• Ultrasound training is still in its infancy in Africa, especially as part of Emergency Medicine training (1, 2)
• Many programs are recently established, support is required to enable them to realize their full potential.
• A viable tool realized by this ongoing pandemic is the strength and versatility of online learning (3)
• These methods have been proven to effectively impart knowledge and skills to even novices in the field of ultrasound and could be useful within African Emergency Medicine (4)

## 1 The evolution of online mentorship

Africa has slowly been adopting ultrasound as a valuable tool in emergency medicine training [1]. Some of these fledgling training programs are short-staffed and require support mainly in the form of mentors who motivate, teach and guide trainees in their journey to becoming competent Point of Care Ultrasound (POCUS) users [2]. Mentorship programs typically aim to: increase exposure to different ultrasound skills and techniques, widen the scope of training as well as ensuring vital competencies are achieved [3]. Locally available resources are used to best achieve these goals such as using phone camera image capture and downloading videos or images from ultrasound devices which can then be shared to mentors to enhance discussions during meetings [4]. These methods solidify tele-ultrasound as an excellent means to provide ongoing ultrasound training by improving ultrasound competency among trainees [5]. Even though the training sessions described were aimed at residents who have already received some ultrasound training, novice users can still participate and gain new skills and knowledge [3].

## 2 Potential gaps in training

Due to limitations such as staff shortages, ultrasound training may not realize its full potential in low resource environments [6]. In addition, ongoing healthcare protocols and measures to curb the spread of COVID 19 have severely hampered hands-on and patient-based bedside ultrasound training. Therefore, a tele-medicine based mentorship program in this age is a practical way of using available resources to enable students to learn while observing infection prevention and control standard operating procedures such as social distancing and working from home. If properly implemented, mentorship programs can help realize the vast potential lying dormant among emergency medicine trainees [4], especially in lower resource areas. While this form of training may be extremely valuable during the pandemic, it also lays crucial foundations for ongoing ultrasound training and mentorship in low resource areas after the resolution of the pandemic.

## 3 The workshop

Mbarara University of science and technology (MUST) in Uganda, in partnership with the University of New York at Buffalo (United States) through the PURE ultrasound mentorship program, collaborated on a 4-h long training session for Emergency Medicine residents at MUST. This training mainly focused on advanced topics in POCUS: Ocular ultrasound, Gallbladder scans, Aortic ultrasound, and soft-tissue imaging as relevant to emergency medicine training. These topics were chosen based on a needs assessment of the residents by simple, informal interviews of multiple residents. The training session was held in a simulation center but could, in principle, be held anywhere, even at home. The aim of the session was to introduce the topics to learners, to have learners visualize how the scans can be done with local setup and lastly perform these scans on volunteers to the satisfaction of the supervisors.

## 4 Recommended steps to successful tele-ultrasound training in low resource environments

1. Needs assessment
  a. A survey of recipients needs to be done to determine the potential deliverables for training sessions. Multiple methods can be employed, from questionnaires and formal research to informal methods.
  b. This particular needs based assessment was done on intermediate ultrasound users who were emergency medicine residents. They had been trained on basics skills and techniques on ultrasound training. Therefore needs analysis and objectives may vary depending on average skills of the participants.
  c. The skills and knowledge levels was assessed during this informal interviews. To ensure basic minimums were reached by all members, reading materials and videos were given prior to the day of the teachings with a basic pre-test to help ensure minimums were met.
2. The sessions were planned according to the availability and accessibility of
  i. Good and reliable internet connection.
  ii. Potential power back up in the event of unstable power supply.
  iii. Multiple laptops or “zoom” capable devices.
  iv. Time variables, primarily if there exists a significant time zone difference between trainers and trainees
  v. Availability of adequate and stable platforms for interactions such as ZOOM and WEBEX among others. Paid subscriptions are preferred so as to reduce distractions and interruptions.
  vi. Portable Ultrasound devices.
3. Stations: Develop stations that represent the most valued skills as determined by your needs assessment. These high yield stations are better off being few with allowance for long discussion and even longer practice times.
4. Educators: Dedicated and motivated trainers should be assigned to each station with the possibility of local duplication of roles. A principal trainer delivers content via telemedicine, and a junior trainer, who is trained to provide appropriate feedback, offers support during practical sessions or as a backup if there is a loss of connectivity.

Learners: for better hands-on experience and increased one-on-one interaction, small groups of 3–5 participants offer the best experience. This is coupled with didactic clinical sessions of up to 30 mins per station. Lastly, for smoother flow, allow advanced users to mix with novice users to enhance the experience.

**Fig. 1:**
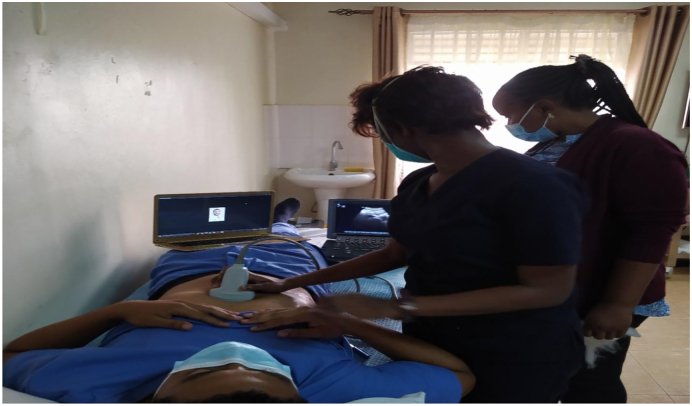
Arrangement for virtual instruction. Both the trainee and the ultrasound screen are visualised by the virtual trainer being performed on a volunteer.

**Fig. 2:**
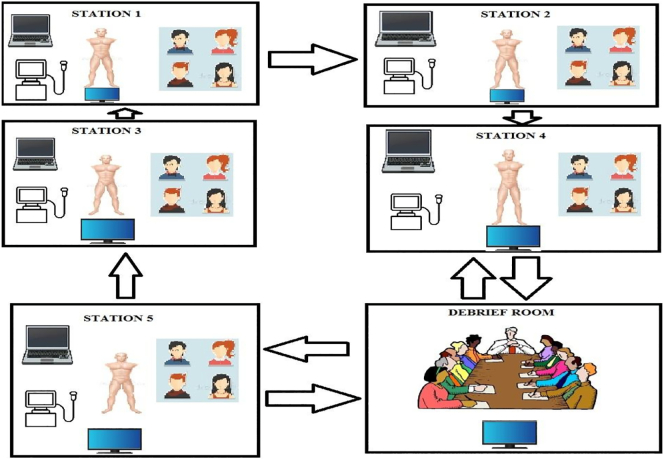
Participant flow chart.

5. Volunteer models: One volunteer per station separate from the participants enhances the delivery of objectives. It also reduces the set-up time for each station and reduces unnecessary interruptions. See Fig. 1 below.

6. Participant flow: It is easier and more feasible to have participants rotate through the stations than trainers. This allows each station to be tailored to each topic, thereby appropriating materials much more efficiently, including ultrasound machines. See Fig. 2 below

Each station was equipped with a laptop with its independent connection, an ultrasound device (e.g. Sonosite or butterfly), a live volunteer and an optional television (Fig. 1). The online supervisor would be on an independent link on zoom, or alternatively have each station have a room on a common zoom link.

Participants can then flow through the rooms and converge in the debrief room. This debrief room can also be used for the initial didactic lectures, breaks or even as a station room.

**Fig. 3:**
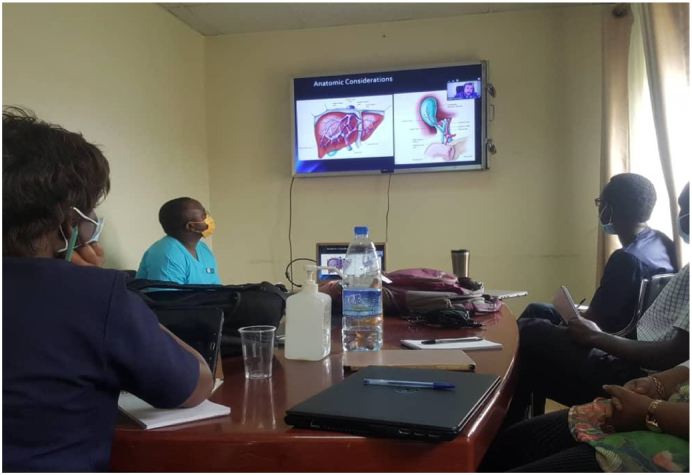
Didactic session.

**Fig. 4:**
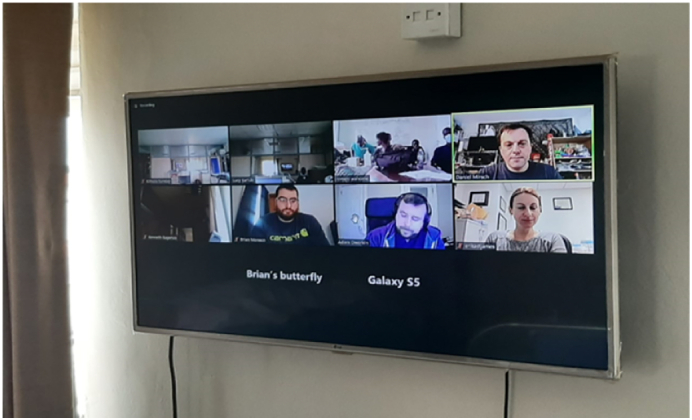
Didactic session and break out rooms.

**Fig. 5:**
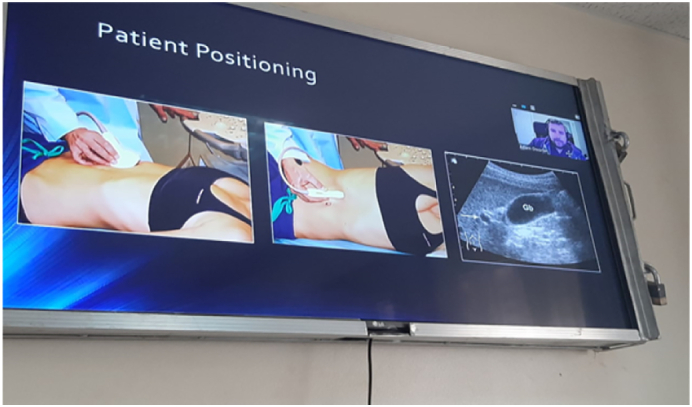
Skill demonstration.

**Fig. 6:**
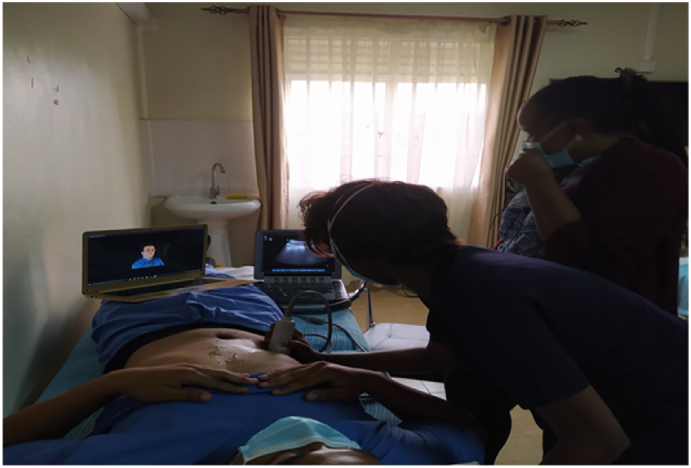
Active trainee perticipation with ultrasound probe positioning being visualised by the trainer.

7. Laptops: Each station should have its own dedicated “zoom capable” device such as a laptop or tablet, with an option to connect to large viewing screens such as TV. The large screens enhance the principal didactic lecture delivery but are not essential in the other stations. Additionally, each zoom device can have its own independent zoom link or can be part of a “ROOM” (it is possible to have multiple separate rooms where users can be allocated, on one common link, see zoom help guide) in a common zoom link. Laptops with good microphones and cameras are an added advantage. See Fig. 3, Fig. 4, Fig. 5, Fig. 6.

8. Network: Typically Mobile phone, airtime based internet is more reliable and stable as compared to office wide networks. Each station should have independent network if possible.
9. Backups: As all machines can fail, backup ultrasounds and backup internet connections should be gathered. This includes keeping all electronic devices fully charged and having modalities such as UPS and power banks.
10. Positioning: The laptop at each station is positioned so that the trainer has both the ultrasound image and the trainee's hand position in view. This view enables more precise directions and instruction. See Fig. 1, Fig. 6, Fig. 7.

**Fig. 7:**
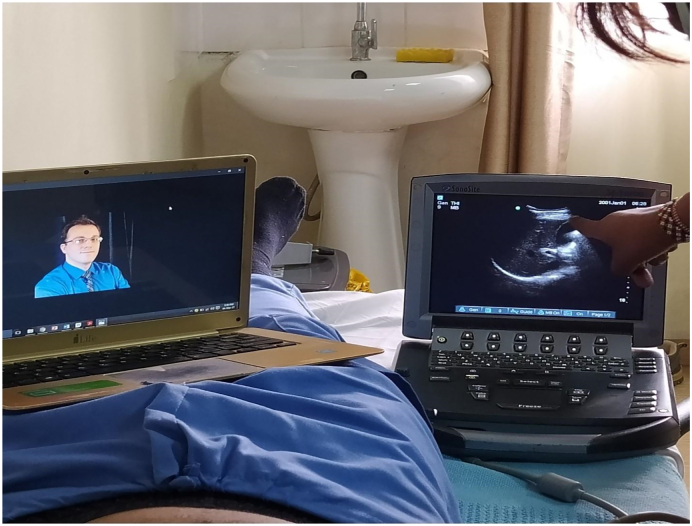
Real time virtual tutor feedback, supplimented by on-site tuitor.
11. Breaks and rest-period: Refreshments and adequate breaks enhance the user experience and improve knowledge retention. See Fig. 8.

**Fig. 8:**
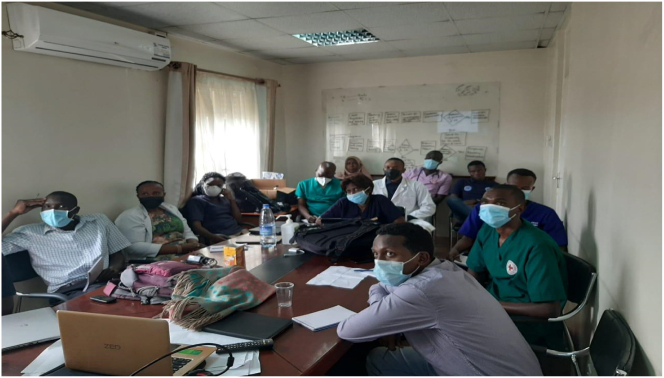
Trainee debriefing.

The session was noted to be very successful, and residents could incorporate the skills learned through the training. This was evidenced by increased confidence in performing the new scans, better reporting, and more ultrasound diagnosis of pathologies aforementioned. This was affirmed by the tutors who noted a great increase in skill and knowledge by the residents evidenced by great scans and improved post test results.

## 5 Conclusion

Tele-ultrasound training across multiple time zones and in low resource environments such as Africa is feasible and can provide a valuable tool in enhancing ultrasound training among emergency medicine residents or other emergency care providers.

## Dissemination of results

The results experienced as part of this workshop have been disseminated institution-wide through formal intra-departmental channels and ongoing in other foreign platforms such as DELTA Africa (an Africa-wide emergency medicine residency support platform), a subsidiary of the Africa Federation of Emergency Medicine. Through DELTA, we are also in the process of reaching other AFEM members.

## Authorship contribution statement

Authors contributed as follow to the conception or design of the work: the design of the conception, development of stations, availing of resources and general organization are as follows: RW and DM contributed 25% each; BM 20% and LK, KB and DH contributed 10% each. All authors approved the version to be published and agreed to be accountable for all aspects of the work.

## Declaration of competing interest

The authors declare no conflict of interest.
